# Pulmonary Embolism Related to Amisulpride Treatment: A Case Report

**DOI:** 10.1155/2013/718950

**Published:** 2013-02-28

**Authors:** Maria Skokou, Philippos Gourzis

**Affiliations:** Department of Psychiatry, University Hospital of Patras, School of Medicine, University of Patras, University Campus, 26504 Rio, Greece

## Abstract

Venous thromboembolism has been associated with antipsychotic drugs, but the underlying mechanisms are largely unknown. Hypotheses that have been made include body weight gain, sedation, enhanced platelet aggregation, increased levels of antiphospholipid antibodies, hyperhomocysteinemia, whereas hyperprolactinemia has recently attracted attention as a potential contributing factor. The highest risk has been demonstrated for clozapine, olanzapine, and low-potency first-generation antipsychotics; however, presently there is no data for amisulpride. In the present paper we describe a case of pulmonary embolism in a female bipolar patient, receiving treatment with amisulpride, aripiprazole, and paroxetine. Although a contribution of aripiprazole and paroxetine cannot completely be ruled out, the most probable factor underlying the thromboembolic event seems to be hyperprolactinemia, which was caused by amisulpride treatment. Increased plasma levels of prolactin should probably be taken into account during the monitoring of antipsychotic treatment as well as in future research concerning venous thromboembolism in psychiatric settings.

## 1. Introduction

Venous thromboembolism (VTE) is a common condition, with an annual incidence of more than 1 per 1000 persons [1]. Deep vein thrombosis (DVT) and pulmonary embolism (PE) are two clinical expressions of VTE, the latter of which, if untreated, is associated with a mortality rate of 30% [2]. Risk factors can be divided in to congenital, such as hereditary thrombophilia, and acquired, including advanced age, obesity, surgery, malignancies, and estrogen therapy [3]. Recent research has focused on increased risk for VTE in psychiatric settings [4].

Psychiatric conditions which have been found dangerous in this regard are physical restraint [5], catatonia [6, 7], and neuroleptic malignant syndrome [8]. Possible underlying mechanisms are immobilization and dehydration in all three conditions, plus vessel injury in the situation of physical restraint, due to heavy resistance of the patient, or fever and rhabdomyolysis in the case of neuroleptic malignant syndrome [4]. Higher risk for VTE has also been demonstrated for specific classes of psychotropic drugs, most consistently for antipsychotics [9–15]. Exact mechanisms have not been elucidated yet, and only recently increased prolactin has been implicated as a potential contributing factor [16]. In the present paper we describe a case of PE most probably related to amisulpride treatment. Amisulpride is a Second Generation Antipsychotic (SGA) with no affinity for serotonin receptors, but with a strong potential for increasing prolactin [17].

## 2. Case Presentation

A 38-year-old woman suffering from mood disorder with psychotic features presented to the emergency department of our hospital complaining of left chest pain. She reported fever during the last three days and mild dyspnea. The vital signs recorded were blood pressure 106/64 mmHg, heart rate 77 beats/min, respiratory rate 22 breaths/min, and body temperature 36.8°C. Body Mass Index (BMI) was 26.5 kg/m^2^. There were some moist rales in the left lower lung on physical examination, the rest of which was negative.

Plasma D-Dimer was 5.19 *μ*g/mL, fibrinogen was 665 *μ*g/dL, and complete blood count revealed hemoglobin 12.0 g/dL, white blood cell count 7.0 K/*μ*L, with 78% neutrophils and platelet count 218 K/*μ*L. Liver and renal function tests as well as cardiac enzymes were normal. Room air blood gas analysis showed hypoxemia and hypocapnia (pO_2_ 76 mmHg, SaO_2_ 95%, PCO_2_ 38 mmHg). Electrocardiogram showed mild sinus tachycardia, and Computed Tomographic Pulmonary Angiography (CTPA) revealed left lobar pulmonary artery thrombosis, regional consolidation, and atelectasis with infection in the left lower lung lobe, and a small pleural effusion. Based on the history and the above results, the diagnosis of pulmonary embolism was established. The patient was admitted in the Department of Pathology and received heparin intravenously at the dose of 24000 IU daily, which was switched to acenocoumarol after three days. She was also treated with levofloxacin 800 mg/d intravenously. As her condition improved, she was discharged from hospital after ten days, on acenocoumarol, which she continued taking for six months, with International Ratio (INR) 2.5–3.0.

The patient was a housewife with three children and had suffered her first major depressive episode, severe with psychotic features, approximately two years before. She then reported depressed mood, suicidal ideation, fatigue, severe anxiety, panic attacks, insomnia, loss of body weight, irritability, ideas of reference, and persecution. She was started on amitriptyline 20 mg/d and perfenazine 8 mg/d by her physician, achieving only partial remission of her symptoms, since her panic attacks remained. One year later she visited the outpatient unit of the Psychiatry Department of our hospital, where her treatment was gradually switched to paroxetine, 30 mg/d, amisulpride, 400 mg/d, and alprazolam, 1 mg/d, until full remission was achieved. About five months later, the patient complained of amenorrhea. Prolactin was found to be high, 92 ng/mL, and amisulpride was gradually switched to aripiprazole, 30 mg/d. By the time she was admitted to the Department of Pathology with PE, amisulpride had already been discontinued for a period of one month, but her prolactin was still high, 65 ng/mL. There was no history of recent surgery or trauma, peripheral vascular disease, cancer or cardiovascular disease, she was not receiving oral contraceptives and her physical activity was normal. She did not use illicit drugs or alcohol, and she smoked 10 cigarettes per day. A full work up for coagulopathy (factor V Leiden, prothrombin deficiency, protein C and S deficiency, antiphospholipid antibodies, activated protein C resistance, elevated factor VIII, and hyperhomocysteinemia) did not show any abnormalities.

The patient was followed up for another three years, during which she demonstrated hypomanic episodes, so the diagnosis of bipolar disorder type II was established. Her treatment was modified to lamotrigine, 300 mg/d, aripiprazole 15–30 mg/d, lorazepam 1–3 mg/d, and paroxetine 20–40 mg/d—apart from the hypomanic phases. Her prolactin measures were normal, and she did not suffer another thromboembolic episode during the follow-up period.

## 3. Discussion

The absence of major congenital or acquired risk factors for PE in the present case points to drug therapy as the most probable factor underlying the thromboembolic event in our patient. Antipsychotic treatment has been linked to higher risk for VTE by most studies [4]. In a most recent report [18], both typical and atypical antipsychotics were associated with higher risk for PE, depending on the specific drug used; highest odds ratio was found for clozapine (OR = 1.47), followed by ziprasidone, risperidone, and olanzapine. On the other hand, aripiprazole and quetiapine were not found to increase the risk [18]. There are also case reports associating olanzapine [19, 20], risperidone [21], and sertindole [22] with higher risk for VTE, but, to our knowledge, no data for amisulpride has been reported.

Underlying mechanisms leading to VTE in the context of antipsychotic treatment are not fully understood. It has been suggested that platelet aggregation is enhanced via 5HT2A antagonism by SGAs, or due to hyperhomocysteinemia [13]. Moreover, typical antipsychotics, especially phenothiazines, and clozapine, have been implicated in the induction of antiphospholipid antibodies [23]. Hyperprolactinemia has been recently found to cause Adenosine-Diphosphate-(ADP-) stimulated platelet activation [16].

As plasma prolactin levels of the patient were elevated at the time of hospital admission, it seems that amisulpride related hyperprolactinemia was the most probable factor contributing to PE in this patient. On the other hand, the fact that she was also taking aripiprazole, paroxetine, and alprazolam at that time should be taken into account too, particularly because aripiprazole, unlike amisulpride, exhibits 5-hydroxytryptamine-2A (5-HT2A) antagonism [17]. Nonetheless, aripiprazole has been reported not to raise the risk for VTE [18]. The same is suggested for antidepressants [24, 25], except for one study [26] and certain case reports [27–29], in support of some association with VTE. On the other hand, Selective Serotonin Reuptake Inhibitors (SSRIs) are much more often implicated in bleeding rather than thrombotic events [30]. Treatment with benzodiazepines has not been linked to VTE [25]. This evidence, together with the fact that the patient continued to receive the above medications for the 3-year follow-up period without another thromboembolic episode, is not in support of a major contribution of drugs other than amisulpride to the PE episode.

Increased prolactin in patients receiving antipsychotics has been a concern most often because of loss of libido, amenorrhea, weight gain, galactorrhea, or even osteoporosis [31]. In the light of the recent findings and as might be suggested by the present paper, monitoring of plasma prolactin levels and increased risk for VTE due to hyperprolactinemia should also be kept in mind by clinicians, as a more acute and potentially fatal adverse event of antidopaminergic agents. Finally, from a research perspective, the inclusion of prolactin measures in future studies dealing with the risk and pathophysiological mechanisms of VTE in the context of antipsychotic treatment could possibly help to disentangle this complex phenomenon.
